# DNA Methylation Pyrosequencing Assay Is Applicable for the Assessment of Epigenetic Active Environmental or Clinical Relevant Chemicals

**DOI:** 10.1155/2013/486072

**Published:** 2013-09-04

**Authors:** Ana-Maria Florea

**Affiliations:** Environmental Toxicology, University of Trier, FBVI, Universitätsring 15, 54296 Trier, Germany

## Abstract

Exposure of cells and organisms to stressors might result in epigenetic changes. Here it is shown that investigation of DNA methylation using pyrosequencing is an alternative for *in vitro* and *in vivo* toxicological testing of epigenetic effects induced by chemicals and drugs. An *in vitro* evaluation of global and CpG site specific DNA methylation upon treatment of cells with chemicals/drugs is shown. Bisulfite genomic sequencing of methylation controls showed high methylation of LINE1 in methylation positive control and low methylation in the negative controls. The CpG sites within the LINE1 element are methylated at different levels. *In vitro* cell cultures show a methylation level ranging from 56% to 49%. Cultures of drug resistant tumor cells show significant hypomethylation as compared with the originating nonresistant tumor cells. The *in vitro* testing of epigenetically active chemicals (5-methyl-2'-deoxycytidine and trichostatin A) revealed a significant change of LINE1 methylation status upon treatment, while specific CpG sites were more prone to demethylation than others (focal methylation). In conclusion, DNA methylation using pyrosequencing might be used not only for testing epigenetic toxins/drugs but also in risk assessment of drugs, food, and environmental relevant pollutants.

## 1. Introduction

Epigenetic factors such as DNA methylation, histone modifications, and microRNAs are able to regulate gene expression and genomic stability [1–3]. CG dinucleotides have unic feature in the mammalian genome: they can associate in CGs cluster regions termed CpG islands [4]. DNA methylation occurs in CpG islands that could be related to transcriptional silencing of genes. Thus, active gens show low methylation in the gene promoter-associated CpG islands, while in downstream-transcribed gene regions and in repetitive regions such as LINEs and SINEs, hypermethylation is found [4–8]. DNA methylation pattern is maintained due to enzymatic activity: DNA methyltransferase (DNMT) family controls *de novo* and maintenance DNA methylation. Therefore, it is possible to modify the DNA methylation status of genes using agents that hinder the function of DNA methylation enzymes [9].

Retrotransposons are mobile elements with the property of moving within a gene via a copy and paste mechanism. Thus, they are responsible for creating genetic variation but also might be the reason for creation disease-causing mutations within the human genome (i.e., insertional mutagenesis, recombination, retrotransposition-mediated and gene conversion-mediated deletion, and 3′ transduction). Active retrotransposable elements include long interspersed elements (LINEs) and short interspersed elements (SINEs) while approximately 0.27% of all human disease mutations are attributable to retrotransposable elements [10]. LINE1 retrotransposons are present in the human genome (estimated 500,000 copies/genome); they disseminate through RNA sequences in DNA sequences after reverse transcription and are then integrated into new genomic loci such as nearby genes. Thus, they are able to epigenetically disrupt the transcriptome and contribute to processes related to tumorigenesis [11].

A large body of toxicological evidence shows that many substances could have an impact on human's health without clear signs of acute or immediate toxicity. In chronic exposure to damaging chemicals (long-term toxicity), it is often found that the chemicals induce changes in the DNA sequence, thus resulting in DNA damage and mutagenesis. Nevertheless, epigenetic research shows that toxic chemicals could also modify the DNA function without changing the sequence, due to, for example, changing the state of DNA methylation, thus inducing deregulated gene expression and genomic instability [1–3, 9]. Thus DNA methylation might play an integral role in toxicity through the hypomethylation of repetitive DNA elements that impacts chromosomal and transcriptional stability of the genome and also as a result of focal hypermethylation, thus resulting in epigenetic silencing of gene expression (DNA methylation and gene expression are inversely correlated) [12, 13].

Long-term toxicological damage could be caused without changing the DNA sequence, thus having important implications on the safety assessment for chemicals, drugs, and food [9]. Epigenetic alterations may be early indicators of genotoxic and nongenotoxic carcinogenic exposure and thus used as biomarkers in the assessment of the carcinogenic potential of environmental chemical and physical agents and might be used in cancer risk assessment [14]. For example, environmental relevant chemicals such as arsenic, cadmium, and bisphenol A have epigenetic modulation property ([3, 15, 16]) but also mixtures of benzene, hydroquinone, styrene, carbon tetrachloride, and trichloroethylene [17]. 

All major human cancers have a large number of genetic alterations and epigenetic abnormalities that might be used as biomarkers for the molecular diagnosis of cancer. The genome-wide loss of methylation at CpG sites of the promoter is a common epigenetic event in malignancies and may play a role in the process of carcinogenesis [14], while changes in promoter DNA methylation and associated gene silencing might be used for cancer therapy (e.g., decitabine and azacitidine) [18]. Thus, investigation of chemical induced changes in DNA methylation status could represent an important indicator for chemical induced risk assessment. 

In this paper it is shown that pyrosequencing is applicable in toxicity tests to investigate the modification in DNA methylation upon exposure to chemicals *in vitro*. Pyrosequencing could be adapted to provide accurate quantifiable data and detailed profiles of DNA methylation patterns underlying, for example, cell cycle regulation, differential gene expression, and epigenetic effects. 

## 2. Material and Methods

Pyrosequencing is a quantitative sequence-based detection technology, adaptable for exploratory and testing work in toxicology and also in pharmacological approaches. Many ready designed assays (PyroMark CpG Assays) are available for pyrosequencing analysis; for example, more than 84,000 assays are available for gene-specific CpG islands including the mouse and rat genomes (Qiagen, Germany; EpigenDX, USA). These assays are created using algorithms that provide specific quantification of CpG methylation. 

Using pyrosequencing for the investigation of DNA methylation has been proofed as highly reproducible and highly sensitive in praxis. Companies are offering to users the possibility of buying ready to use kits for pyrosequencing especially diagnostic relevant biomarkers such as KRAS, MGMT, p16, LINE1, MLH1, and BRAF (Qiagen, Germany; EpigenDX, USA). Although the number of available predesigned ready to buy DNA methylation assays (e.g., PyroMark CpG Assays) has considerably increased in the market, the researcher today might be put in the position that there are no assays available for specific biomarkers. Thus, it is possible to create self-designed assays using commercial software (PSQ assay design) or freely available software (Primer3). 

### 2.1. Cell Culture

Normal human dermal fibroblasts (NHDF) were provided from PromoCell, Germany. The cells were isolated from normal human foreskin from a 10-year old donor and cryopreserved at passage 2 (P2) using serum-free freezing medium, Cryo-SFM (PromoCell, Germany). The cultures were maintained in the incubator at 37°C, 5% CO_2_. The cells were split once they have reached 80–95% confluence by using Trypsin-EDTA (0.025%) (PAA, Germany). After collecting the detached cells in Trypsin inhibitor (PromoCell, Germany) the cells were maintained in cell culture dishes (NUC, Germany). The fibroblast cultures were maintained in serum-free fibroblast culture medium, without phenol red, supplemented with “Supplement Mix” (basic fibroblast growth factor 1 ng/mL and insulin 5 ng/mL) (PromoCell, Germany) as well as antibiotics: penicillin/streptomycin (PAA, Germany). 

HACAT cells (DKFZ, Germany) are *in vitro* spontaneously transformed keratinocytes derived from histological normal skin that grows in DMEM medium (high glucose) supplemented with 2 mM L-glutamine (PAA) and 10% fetal calf serum (PAA, Germany) as well as antibiotics: penicillin/streptomycin (PAA, Germany). The cultures were maintained at 37°C with 5% CO_2_ and splitted as necessary if they reached 80–95% confluence [19].

### 2.2. 5-Methyl-2′-deoxycytidine and Trichostatin A Treatment (A + T2) of HACAT Cells

HACAT cells were plated in 10 cm^2^ culture dishes at 50% confluence and were left over night for attachment. They were treated (*n* = 6) with the demethylating agent 5-aza-2′-deoxycytidine (5-Aza) and the histone deacetylase inhibitor trichostatin A (TSA) (Sigma, Germany). The treatment conditions were done with either the demethylating agent 5-aza-2′-deoxycytidine (1 *μ*M for 72 h) or the histone deacetylase inhibitor trichostatin A (1 *μ*M for 36 h). 

### 2.3. Glioma Cell Lines LNT-229, LN-308, and LN-18

All glioblastoma cell lines were grown under standard condition and as elsewhere described [20]. Dulbecco's modified Eagle's medium (DMEM) was used as basal medium. 10% (v/v) heat-inactivated fetal bovine serum (FCS) and 1% (v/v) penicillin G/streptomycin were added to the medium and cells were incubated at 37°C in a humidified atmosphere containing 5% CO_2_. The cell culture of glioma cell lines and temozolomide (TMZ) resistant cells were elsewhere described [20]. Cell pellets from drug sensitive and drug resistant cell lines were provided for nucleic acid extraction and pyrosequencing [20].

### 2.4. Kidney Carcinoma CKA-CA6 Cells and Human Embryonic Kidney HEK293 Cells

The DNA was kindly provided by PD Dr. Csaba Mahotka (Pathology, HH University Düsseldorf). The DNA extraction was done using the Qiagen DNA extraction kit and the company provided protocol. The cells were grown using standard cell culture procedures. 

### 2.5. Nucleic Acids Extraction and Bisulfite Conversion of the Genomic DNA

Nucleic acid extraction was performed as reported elsewhere [21] using ultracentrifugation and caesium chloride method followed by the phenol/chloroform purification or using the DNA extraction kit from Qiagen, Germany, using the providers protocol. One microgram of genomic DNA was further subject of bisulphite transformation using the EZ DNA Methylation Gold Kit (Hiss Diagnostics, Germany) in order to be used in pyrosequencing reactions.

### 2.6. Primer Design

Long interspersed nuclear element 1 (LINE1 or L1) sequences are highly repeated human retrotransposon sequences and constitute about 17% of the human genome. DNA methylation within the promoter region of human LINE1 elements is important for maintaining transcriptional inactivation and for inhibiting transposition [22]. Genome-wide losses of DNA methylation within the promoter region of human LINE1 elements have been regarded as a common epigenetic event in malignancies and may play crucial roles in carcinogenesis [11].This methylation assay amplifies a region of the LINE1 element and serves as a marker for global methylation. PyroMark LINE1 from Qiagen, Germany, has been used following the company recommendations and elsewhere described [20]. CpG sites are located in positions 331 to 305 of LINE1 (GenBank accession number X58075).

**Figure 1:**
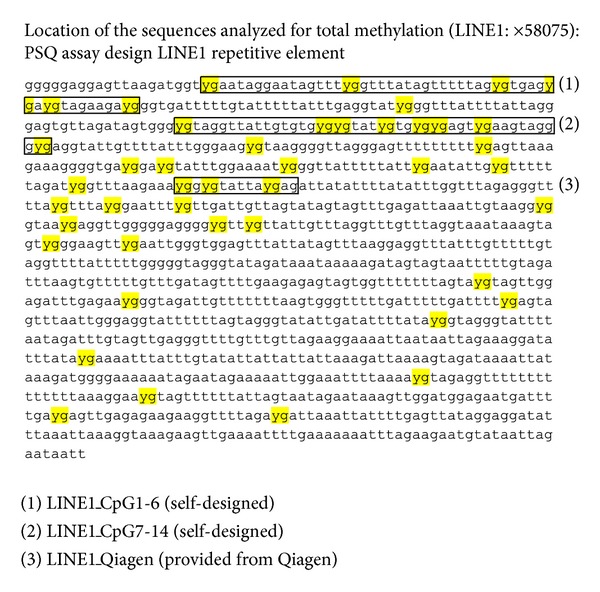
Long interspersed nuclear element 1 (LINE1 or L1) sequences are highly repeated human retrotransposon sequences. Here, the LINE1 GenBank accession number X58075 is shown after *in silico* bisulphite conversion; the location of the investigated primers are shown.

Self-designed assays were obtained using the PSQ assay design. The sequence of LINE1 element was obtained using RefSeq (GenBank accession number X58075). The location of each primer combination is shown in Figure 1. With the help of Microsoft Word (Microsoft, USA) the LINE1 sequence was *in silico* bisulfite converted (all CG => YG; all C => T) and pasted in the primer design software. The result of the primer design is shown in Tables 1 and 2. The LINE1_CpG1-6 is located at the beginning of the sequence (X58075) and is spanned over 6 CpG sites while LINE1_CpG7-14 is located after LINE1_CpG1-6 and is spanned over 8 CpG sites.

### 2.7. Pyrosequencing

For pyrosequencing the PyroMark LINE1 kit (Qiagen, Germany) was used following the company recommendations. In brief, each PCR mix contained 1× PCR buffer, 1.5 mmol/L of MgCl_2_ (final concentration) (Qiagen), 0.2 mol/L of each dNTP (Biobudget, Germany), 1 *μ*L of forward and reverse primer (10 pmol/L of each PCR primer), 2 U of HotStar Taq Polymerase (Qiagen), and 2 *μ*L of bisulfite treated template DNA in a total volume of 50 *μ*L. 

PCR conditions were as follows: initial denaturing at 95°C for 15 minutes; 45 cycles of 95°C for 20 seconds, 50°C for 20 seconds, and 72°C for 20 seconds; and final extension at 72°C for 5 minutes giving an amplicon length of 146 bp. The PCR product was checked by 1% agarose gel electrophoresis (data not shown). The biotinylated PCR product was purified to single-stranded DNA to be the template in a pyrosequencing reaction, as recommended by the manufacturer using the Pyrosequencing Vacuum Prep Tool (Qiagen, Germany). 

Forty microliters of biotinylated PCR product were immobilized on streptavidin coated sepharose beads. Thereafter, the sepharose beads containing the immobilized PCR product were purified, washed, denatured using a 0.2 M NaOH solution, and washed again. Then, 0.3 *μ*M pyrosequencing primer was annealed to the purified single-stranded PCR product and pyrosequencing was performed by PyroMark Q24 (Qiagen, Germany). The sequence to analyze was TTYGTGGTGYGTYGTTT while the dispensation order was GCTCGTGTAGTCAGTCG [20].

**Figure 2:**
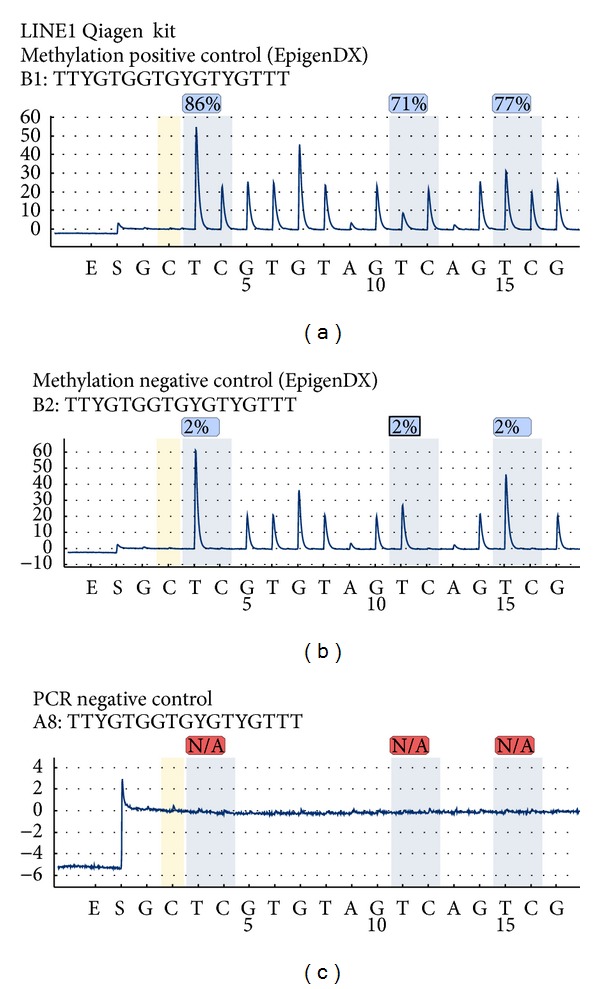
Human methylation positive and negative controls (EpigenDX, USA) and PyroMark LINE1 kit (Qiagen, Germany).

Similarly it was proceeded with the self-designed primers LINE1_CpG1-6 and LINE1_CpG7-14. Primers were ordered at Eurofins (Germany) and reassembled as recommended by the provider. For PCR reaction the HotStar Taq Polymerase from Qiagen was used. PCR conditions: 95°C-5 min. (1 cycle); 95°C-1 min., 57°C-1 min., 72°C-1 min. (50 cycles); 72°C-5 min. (1 cycle); 4°C ∞. The assays were proofed using human methylation positive and negative controls as seen in Figure 2 (ordered at EpigenDX, USA). The pyrosequencing was done as recommended by the provider using the PyroGold Reagents 5 × 24 (Qiagen, Germany) and the PyroMark Q24 Qiagen (Germany) and as described in detail above.

### 2.8. Statistics

Statistical analysis was done comparing control versus each condition while Student's *t*-test was applied where *P < 0.05.

## 3. Results

### 3.1. The Setup and the Quality Control of a DNA Methylation Pyrosequencing Assay

To make sure that a specific DNA methylation assay functions in a given laboratory condition the methylation PCR reaction followed by pyrosequencing should be tested. Known methylation status DNA samples such as positive and negative methylation controls are used. They are available for purchase at many providers (e.g., EpigenDX, USA; Millipore, Germany) while mouse and rat methylation controls are available as well. For this paper are shown the results obtained with the human methylation positive and negative controls bought at EpigenDX, USA (Figure 2) and PyroMark LINE1 kit (Qiagen, Germany). 

As described in the Material and Methods, for each test reaction was used 200 ng bisulphite transformed DNA for (Figure 2 from (a) to (c)) (1) methylation positive control; (2) methylation negative control; (3) PCR negative control. The establishment of the PCR negative control is very important in order to check for (i) the contamination of the PCR reaction, (ii) specificity of the primers, and (iii) size of the PCR product. The PCR reaction was assembled as described at the Material and Methods. After quality check on 1-2% agarose gel (showing one clear PCR band) the PCR product is purified with the Pyrosequencing Vacuum Prep Tool (Qiagen, Germany) and proceeded with the pyrosequencing. 

Typical good functioning pyrosequencing assay is shown in Figure 2 (PyroMark LINE1 kit (Qiagen, Germany)). In this case, the LINE1 sequence that was assessed by pyrosequencing has three CpG sites (see location in Figure 1). Over each of the CpG site the found methylation level and the quality of the obtained result (red = bad quality, yellow = pass quality, and blue = good quality) are shown. The pyrogram shows in addition the well analyzed and the sequence to analyze. Furthermore, on the *x*-axis the nucleotides injected by the pyrosequencer are shown while on the *y*-axis the luminescence detected by the LCD camera is shown. 

In brief, in the shown pyrograms (Figure 2), the “E” represents the moment when the enzyme was add to the reaction followed by the substrate “S” that is documented by a small spike. The pyrosequencer added a “G” that is not present in the sequence (as in build reaction control); thus, no spike was observed. Then the nucleotide “C” is added to the reaction that represents the bisulphite transformation control; in this case the bisulphite transformation is complete because no signal was detected by the pyrosequencer. The actual sequence to analyze starts with a TT (that is double as high as the single T) followed by our methylated cytosine here marked with Y. The percentages for methylation are the result of the calculated ratio: thymine versus methylated cytosine. The pyrosequencing continues with adding one by one the nucleotides (here: G, T, GG etc.); if the nucleotide fits the sequence a light signal is detected, if not no light signal is detected. 

In the methylation positive control it is shown that each of the CpG sites has a high level of methylation, for example, CG1 = 86%; CG2 = 71%; CG3 = 77%. In the methylation negative control it is shown that each of the CpG sites has a low level of methylation, for example, CG1 = 2%; CG2 = 2%; CG3 = 2%. If one compares in the pyrogram the spike present for the detection of the methylated cytosine in the methylation negative control does not practically exist. Furthermore, the PCR negative controls show no specific spikes whatsoever of the nucleotides are injected in the pyrosequencing reaction; thus, this pyrosequencing assay is specific. 

### 3.2. Different Cell Lines Show Different Methylation Status of the LINE1 Element

**Figure 3:**
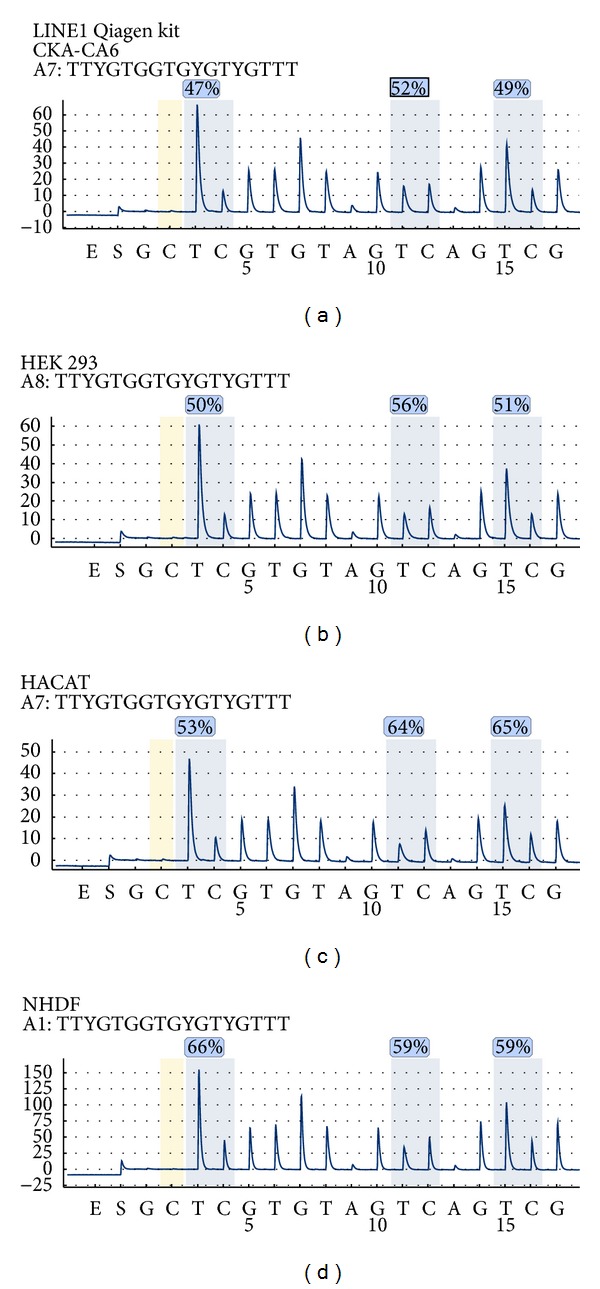
Established tumor, nontumor, and primary cell lines investigated for their intrinsic methylation level of the LINE1 element (Qiagen, Germany): CKA-CA6 kidney carcinoma; HEK293 human embryonic kidney cells, spontaneously immortalized human keratinocytes (HACAT), and primary *in vitro* cultures of normal human dermal fibroblasts (NHDF).

**Figure 4:**
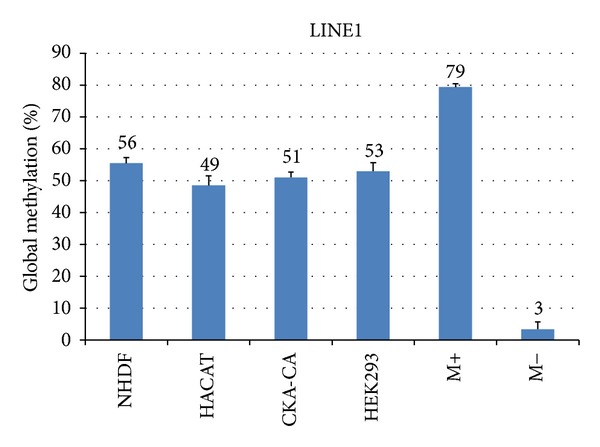
Quantitation of intrinsic methylation level of the LINE1 element (Qiagen, Germany) in established tumor, nontumor, and primary cell lines using several measurement replicates (*n* = 3–6) and the correspondent methylation controls (Student's *t*-test, *P < 0.05).

Well-established cell lines were investigated for their intrinsic methylation level of the LINE1 element. In Figure 3, from (a) to (d), the pyrosequencing of LINE1 element (Qiagen, Germany), a kidney carcinoma cell line is shown (CKA-CA6), followed by human embryonic kidney cells (HEK293), spontaneously immortalized human keratinocytes (HACAT), and primary *in vitro* cultures of normal human dermal fibroblasts (NHDF). The three investigated CpG sites show different levels of methylation in each cell line investigated. Also the methylation level was slightly different in each cell line (Figure 4). The highest level of methylation was found for NHDF that was about 56% (*n* = 5) followed by 53% for HEK293 (*n* = 3), 51% for CKA-CA (*n* = 3), and 49% for HACAT cells (*n* = 5). The measured methylation value of methylation positive control was 79% while for methylation negative control was 3%. 

### 3.3. Drug Sensitive and Drug Resistant Glioma Cell Lines Show Different Methylation Status of the LINE1 Element

**Figure 5:**
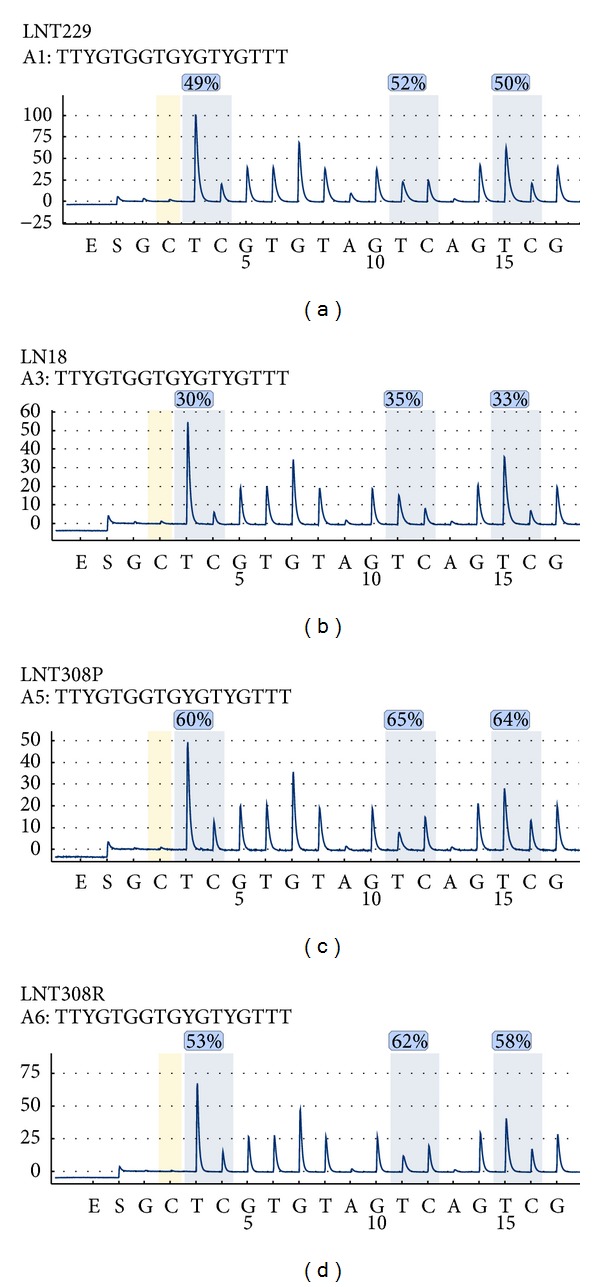
Glioma cell lines were investigated for the methylation level of LINE1 element (Qiagen, Germany) for LNT 229, LN 18, and LNT 308. Temozolomide resistant strains of same cell lines that were obtained with treatment of increasing concentrations of the drug; exemplarily depicted is LNT 308 P (drug sensitive) and LNT 308 R (drug resistant).

**Figure 6:**
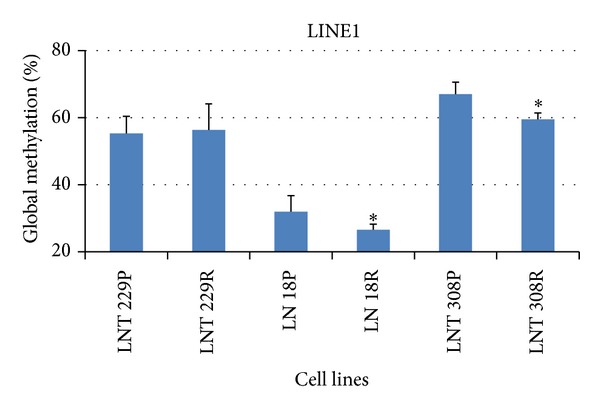
Quantitation of intrinsic methylation level of the LINE1 element (Qiagen, Germany) in Temozolomide sensitive and resistant glioma cell lines using several measurement replicates (*n* = 3–6) and the correspondent methylation controls (Student's *t*-test, *P < 0.05).

Three glioma cell lines were investigated for the methylation level of LINE1 element as shown in Figure 5 from (a) to (d) for LNT 229, LN 18, and LNT 308. The LINE1 methylation levels (Qiagen, Germany) varied dramatically between the three cell lines (Figure 6); the highest methylation was observed in LNT 308 cells followed by LNT 229 and LN 18. In the temozolomide resistant strains of same cell lines that were obtained with treatment of increasing concentrations of the drug, a decrease in the global methylation was observed, as exemplarily depicted for LNT 308 P (drug sensitive) and LNT 308 R (drug resistant) (Figure 5(d)). Overall, the decrease in global methylation was significant for LN 18 R and LNT 308 R but not for LNT 229 P (Student's *t*-test, *P < 0.05). This is an indication not only that genes responsible for drug resistance might be activated but also that LINE1 element might play an important role in the temozolomide drug resistance of glioblastoma [20].

### 3.4. The Use of Self-Designed Pyrosequencing Assays in the Evaluation the Modifications in DNA Methylation of Epigenetic Regulating Chemicals/Drugs

**Figure 7:**
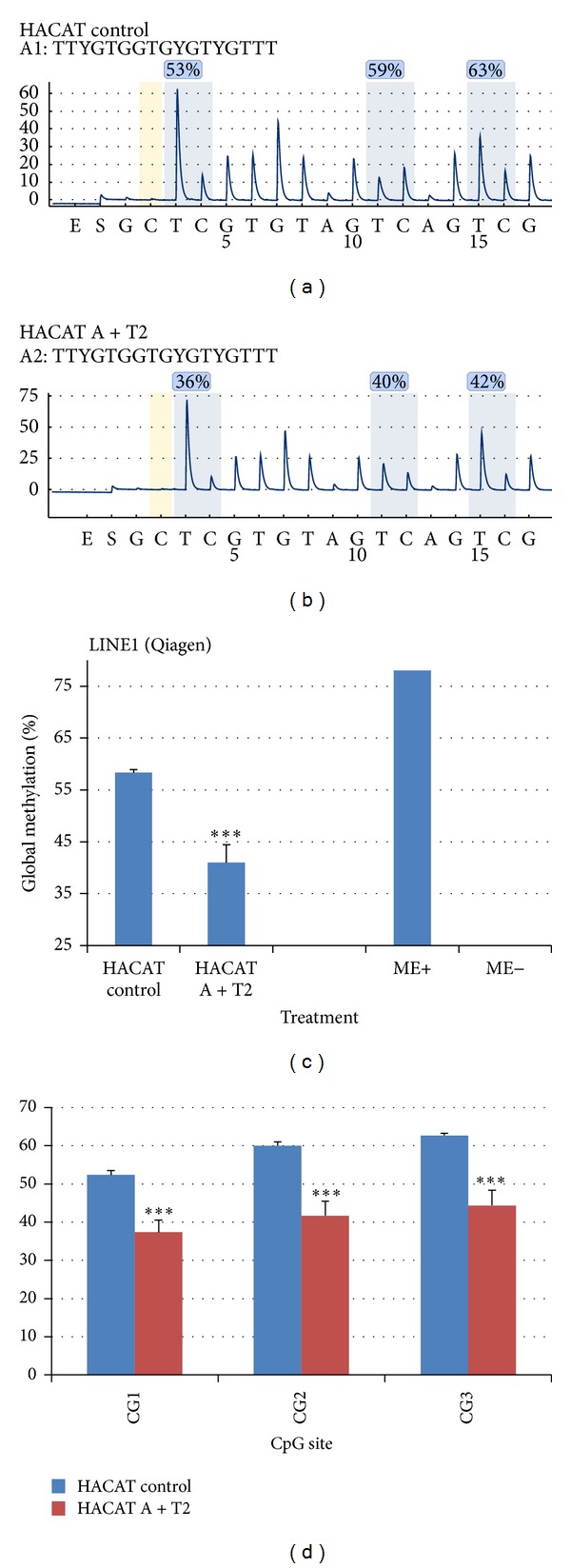
Assessment of HACAT cells methylation after *in vitro* chemical treatment using the self-designed assays (PSQ primer design, Qiagen, Germany). HACAT cells were treated *in vitro* with DNA demethylating agent 5-aza-2′-deoxycytidine (A) (1 *μ*M for 72 h) and the histone deacetylase inhibitor trichostatin A (T2) (1 *μ*M for 36 h).

Here the similarity between the commercial available LINE1 element kit (Qiagen, Germany) and the self-designed assays (PSQ primer design, Qiagen, Germany) was investigated (Figure 1 and Tables 1 and 2). HACAT cells were treated *in vitro* with DNA demethylating agent 5-aza-2′-deoxycytidine (A) (1 *μ*M for 72 h) and the histone deacetylase inhibitor trichostatin A (T2) (1 *μ*M for 36 h) as shown in the Material and Methods. The results obtained in the pyrosequencing of LINE1 element kit (Qiagen, Germany) are shown in Figure 7. 

Observe that in the HACAT control cultures the methylation level of each CpG site was 53%, 59%, and 63%, levels that decreased significantly (Student's *t*-test, *P < 0.05) in HACAT A + T2 treated cells to 36%, 40%, and 42%. The total methylation of the 3 CpG sites decreased significantly (Student's *t*-test, *P < 0.05) HACAT A + T2 treated cells; however the decrease was not as high as compared to the methylation positive and negative controls. Also the significant (Student's *t*-test, *P < 0.05) decrease in methylation HACAT A + T2 treated cells was identified in each of the CpG sites of the LINE1 element kit (Qiagen, Germany). 

**Figure 8:**
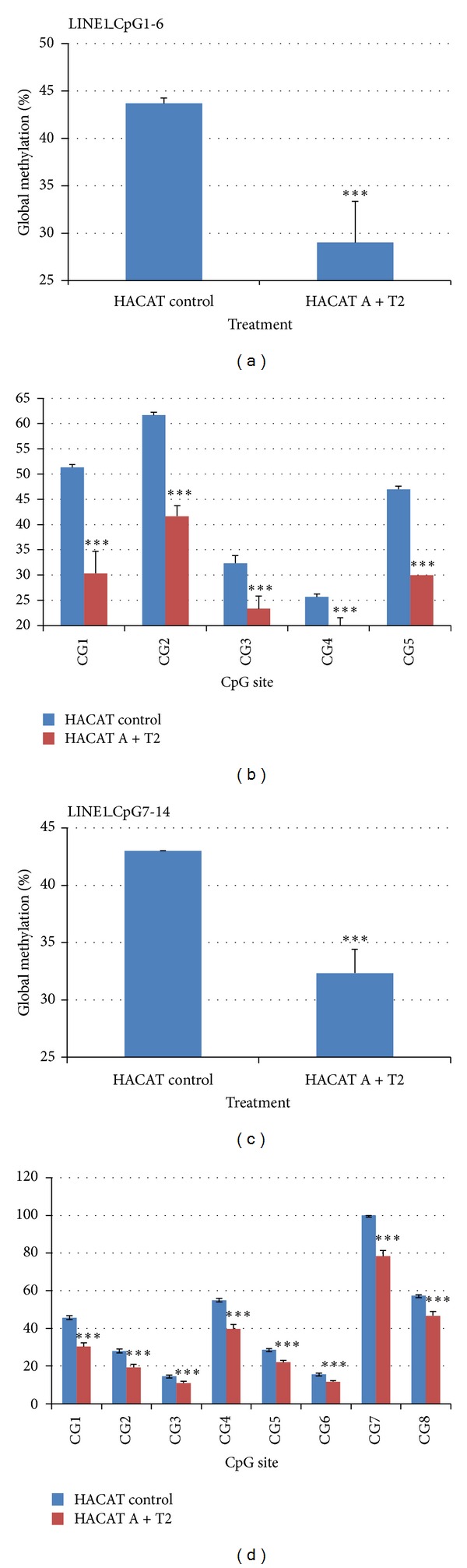
Assessment of HACAT cells methylation after *in vitro* chemical treatment using LINE1 element kit (Qiagen, Germany). HACAT cells were treated *in vitro* with DNA demethylating agent 5-aza-2′-deoxycytidine (A) (1 *μ*M for 72 h) and the histone deacetylase inhibitor trichostatin A (T2) (1 *μ*M for 36 h).

Similar results were obtained with the self-designed assays (PSQ primer design, Qiagen, Germany) (Figure 1 and Tables 1 and 2). The pyrosequencing assay Line1_CpG1-6 spanned over 5 CpG sites located at the beginning of the LINE1 element. As seen with previous assay the significant decrease in total methylation (over all 5 CpG sites) was documented in Figure 8(a); however each CpG site showed decreased methylation level that was at least as strong as observed for the pyrosequencing with the LINE1 element kit from Qiagen, Germany. Nevertheless the decrease in the LINE1 methylation was different in the CpG site; a fact that might underline that specific CpG sites could be preferentially targeted by the epigenetic regulators (chemicals and drugs). 

The pyrosequencing assay Line1_CpG7-14 spanned over 8 CpG sites located at the beginning of the LINE1 element, after Line1_CpG1-6 but before the primers of LINE1 element kit, Qiagen, Germany. Also here, the significant decrease in total methylation (over all 8 CpG sites) was documented in Figure 8(d). Each CpG site showed decreased methylation level that was less strong as observed for the pyrosequencing with the LINE1 element kit from Qiagen, Germany, and Line1_CpG1-6. As well, the decrease in the LINE1 methylation was different in each CpG site; a fact that might underline that specific CpG sites could be preferentially targeted by the epigenetic regulators (chemicals and drugs).

## 4. Discussion

Toxic agents that are not mutagenic could cause stable adverse phenotypic changes if they interfered with epigenetic processes. DNA methylation in regulatory regions such as promoters and enhancers could silence gene expression, and an inverse correlation between gene expression and DNA methylation in promoters was proposed. The process of DNA methylation has therapeutic and toxic implications. Agents that interfere with DNA methylation processes could be used to remove deleterious DNA methylation therapeutically or on the other hand could introduce DNA methylation aberration with toxic consequences. Nevertheless, DNA methylation inhibitors were introduced into clinical practice for anticancer treatments [9, 20].

### 4.1. Assessment of DNA Methylation as Toxicological Evidence

Adverse health effects induced in humans upon exposure to environmental components (particulate matter and ozone) and pollutants (arsenic, cadmium, bisphenol A, and paraquat) might cause changes the epigenome [3, 15, 16, 23, 24]). Thus, epigenetics is an important mechanism in the ability of environmental chemicals to influence health and disease [3]. 

In this paper it is shown that LINE1 element can be used as indicator not only for epigenetic modifying chemicals for global methylation but also for focal methylation. Commercial available kits and self-designed assays can be used. *In vitro* available and established cell lines are suitable for investigation of stressors that affect DNA methylation while epigenetic modifying drugs are able to change the methylation profiles *in vitro*. These findings are supported by previous publications such as that of Tabish et al., 2012, that shows the global DNA methylation changes in human lymphoblastoid (TK6) cells (*in vitro*) in response to 5 direct and 10 indirect-acting genotoxic agents. The authors show the effect of exposure of 5-methyl-2′-deoxycytidine between control and exposed cultures but also to benzene, hydroquinone, styrene, carbon tetrachloride, and trichloroethylene and they concluded that changes in global DNA methylation are an early event in response to agents traditionally considered as genotoxic [17]. 

### 4.2. Assessment of DNA Methylation in Clinical Practice

Modification in DNA methylation could be used as indicator for increased occupational risk in workers. Hints in this direction was given in a recent study of Godderis et al., 2012, where it was found that global DNA hypermethylation was found in the lymphocytes of solvent-exposed worker population compared with the referents (*P* = 0.001, *r* = −0.544) and was negatively associated with the exposure [25]. Furthermore, DNA methylation could be used as early marker in the development of serious diseases such as cancer [26] and Parkinson's disease [24]. Additionally, it could be used in clinical diagnostics: emerging research demonstrates that DNA methylation is responsible for cellular differentiation and, when measured in whole peripheral blood, serves not only to distinguish cancer cases from controls [26] but also to distinguish between the drug sensitive and drug resistant cells.

## Figures and Tables

**Table 1a:** (a)

Primer set 1	Score: 76
General warnings	

**Table 1b:** (b)

⇀	F1	GGGAGGAGTTAAGATGGT
*⥞*	R1	ATAAACCCCATACCTCAAA
→	S1	GGGAGGAGTTAAGATGGT

**Table 1c:** (c)

	PCR product	Forward PCR primer, F1	Reverse PCR primer, R1	Sequencing primer, S1
Length, bp	109	18	19	18
Position, 5′-3′		3-20	111-93	3-20
Warnings				
Tm, °C		60.2	60.9	53.1
% GC	33.0	50.0	36.8	50.0
Sequence to analyze	YGAATAGGAATAGTTTYGGTTTATAGTTTTTAGYGTGAGYGAYGTAGAAGAYGGGTGATTTTTGTATTTTTATTTGA			

**Table 2a:** (a)

Primer set 1	Score: 81
General warnings	

**Table 2b:** (b)

⇀	F1	GGTTTATTTTATTAGGGAGTGTTA
*⥞*	R1	AAAAAAAAACTCCCTAACC
→	S1	AGGGAGTGTTAGATAGTGG

**Table 2c:** (c)

	PCR product	Forward PCR primer, F1	Reverse PCR primer, R1	Sequencing primer, S1
Length, bp	127	24	19	19
Position, 5′-3′		105-128	231-213	118-136
Warnings				
Tm, °C		61.9	59.3	50.8
% GC	37.0	29.2	31.6	47.4
Sequence to analyze	GYGTAGGTTATTGTGTGYGYGTATYGTGYGYGAGTYGAAGTAGGGYGAGGTATTGTTTTATTTGGGAAG			
